# Prevalence of viral respiratory tract infections in acutely admitted and ventilated ICU patients: a prospective multicenter observational study

**DOI:** 10.1186/cc14184

**Published:** 2015-03-16

**Authors:** F Van Someren Greve, KF Van der Sluijs, R Molenkamp, AM Spoelstra-de Man, OL Cremer, RB De Wilde, PE Spronk, MD De Jong, MJ Schultz, NP Juffermans

**Affiliations:** 1 Academic Medical Center, Amsterdam, the Netherlands; 2 VU Medical Center, Amsterdam, the Netherlands; 3 University Medical Center Utrecht, the Netherlands; 4 Leiden University Medical Center, Leiden, the Netherlands; 5 Gelre Hospitals, Apeldoorn, the Netherlands

## Introduction

The prevalence of viral respiratory tract infections in critically ill patients is uncertain, as well as the optimal diagnostic method to detect these. The aim of this study was to assess the prevalence of viral respiratory tract infections in mechanically ventilated patients, in both the upper and lower respiratory tract.

## Methods

A prospective observational study was performed in five ICUs in the Netherlands. From September 2013 to April 2014, consecutive acutely admitted, mechanically ventilated patients were included, regardless of diagnosis at admission. Nasopharyngeal (NP) swabs and tracheal aspirates (TA) were collected at intubation, and were tested via multiplex RT-PCR for the following viruses: influenza A and B, parainfluenzaviruses, RSV, human metapneumoviruses, bocaviruses, coronaviruses, rhinoviruses, enteroviruses, parechoviruses and adenoviruses. Viral DNA/RNA copies were expressed by crossing-point (cp) values.

## Results

In total, 1,499 patients were included, of whom 265 patients (18%) had a viral respiratory tract infection with at least one virus. In 17 patients, two viruses were found; two patients had an infection with three viruses. The most prevalent was parainfluenzavirus-3 (5.7%); 17 patients (1.1%) had an infection with influenza. The lowest prevalence of viral infections occurred in September (12%), the highest in October and February (both 26%). Of the patients tested positive in TA, only 46% also tested positive in NP. The median cp values were not significantly different between TA and NP swabs (31.1 vs. 31.6, *P *= 0.75).

## Conclusion

The prevalence of viral respiratory tract infections is high in unselected ICU patients. Testing tracheal aspirate in combination with nasopharynx greatly increased detection of viruses, and yields similar cp values. Whether these viral infections are associated with prolonged mechanical ventilation and worse outcomes remains to be determined.

